# Dynamic changes in hs-CRP and risk of all-cause mortality among middle-aged and elderly adults: findings from a nationwide prospective cohort and mendelian randomization

**DOI:** 10.1007/s40520-024-02865-w

**Published:** 2024-10-26

**Authors:** Zhonghai Wang, Feng Xiong, Quanbo Zhang, Han Wang

**Affiliations:** 1 Department of Cardiology, Affiliated Hospital of Southwest Jiaotong University, The Third People’s Hospital of Chengdu, Chengdu, Sichuan China; 2 Department of Geriatrics, North Sichuan Medical College, Nanchong, Sichuan China

**Keywords:** Longitudinal trajectories, High-sensitivity c-reactive protein, All-cause mortality, Mendelian randomization, SHapley additive exPlanations

## Abstract

**Introduction:**

The general population experiences mortality rates that are related to high levels of high-sensitivity C-reactive protein (hs-CRP). We aim to assess the linkage of longitudinal trajectories in hs-CRP levels with all-cause mortality in Chinese participants.

**Methods:**

We utilized data from the China Health and Retirement Longitudinal Study (CHARLS). The exposures were dynamic changes in the hs-CRP and cumulative hs-CRP from 2012 to 2015, and the outcome was all-cause mortality. All participants were categorized into four trajectories according to hs-CRP levels. Multivariable logistic regression analysis, adjusted for potential confounders, was employed to evaluate the relationship of different trajectories of hs-CRP with mortality risk. A two-sample Mendelian randomization (TSMR) method and SHapley Additive exPlanations (SHAP) for identifying determinants of mortality risk were also employed.

**Results:**

The study included 5,445 participants with 233 deaths observed, yielding a mortality proportion of 4.28%. Compared to individuals maintaining low, stable levels of hs-CRP (Class 1), individuals with sustained elevated levels of hs-CRP (Class 4), those experiencing a progressive rise in hs-CRP levels (Class 2), or those transitioning from elevated to reduced hs-CRP levels (Class 3) all faced a significantly heighted death risk, with adjusted Odds Ratios (ORs) ranging from 2.34 to 2.47 across models. Moreover, a non-linear relationship was found between them. Further TSMR analysis also supported these findings. SHAP showed that hs-CRP was the fifth most important determinant of mortality risk.

**Conclusions:**

Our study shows all-cause mortality increases with dynamic changes in hs-CRP levels among middle-aged and elderly adults in China, and cumulative hs-CRP shows an L-shaped relationship with all-cause mortality.

**Supplementary Information:**

The online version contains supplementary material available at 10.1007/s40520-024-02865-w.

## Introduction

Chronic inflammation plays a crucial role in the development and progression of many chronic illnesses, including cardiovascular diseases, diabetes, and cancers [1–3]. High-sensitivity C-reactive protein (hs-CRP), recognized as a sensitive indicator of inflammation, can rise dramatically in response to bodily inflammation. Additionally, owing to its straightforward measurement and economical nature, CRP has emerged as a crucial instrument for clinically assessing inflammatory states and predicting disease risk [2, 4]. The principal biological role of CRP is characterized by its capacity to identify and bind to pathogens, apoptotic cells, and other potential inflammatory mediators. Nevertheless, contemporary investigations have unveiled that its function extends beyond mere inflammation indication, it may actively contribute to the inflammatory response, impacting vascular endothelial function and escalating atherosclerosis progression, which in turn amplifies the risks of incidence and mortality from cardiovascular diseases [5]. An extensive array of research efforts has been directed towards deciphering the linkage between CRP and the overall mortality risk, with their findings indicating that an increase in CRP is linked with a heighted risk of all-cause mortality, particularly among individuals with cardiovascular diseases and specific types of cancers [6–8]. For instance, a comprehensive meta-analysis that included 22 studies with a total of 484,821 participants indicated that compared to the low CRP group, the pooled relative risks for all-cause mortality in the moderate and high CRP groups were 1.30 and 1.75, respectively [8]. These findings also elucidated a nonlinear relationship of baseline CRP levels and mortality. Furthermore, a series of studies have discovered the interplay between CRP and mortality risk may be influenced by various factors such as genetic background, lifestyle, and chronic disease status [9, 10]. Consequently, the nature of their association necessitates further comprehensive inquiry to unravel the underlying mechanisms and to assess its clinical implications.

The traditional CRP techniques exhibit limited sensitivity in detecting low-grade inflammatory states, and hs-CRP technology currently enables precise quantification of CRP, even at lower concentrations, which is crucial for detecting inflammatory states and predicting disease risks. Despite the widespread use of baseline CRP levels in research, their limitations are becoming increasingly apparent. Recent evidences have suggested that dynamic alterations in CRP levels, including temporal trends and cumulative effects, can offer a more accurate representation of an individual’s inflammatory status and health risks [6, 7]. Nevertheless, the majority of studies assessing their association have primarily focused on high income countries. Given consistent burden of chronic diseases among adults, particularly in low-and middle-income countries like China, exploring the relationship of dynamic changes in hs-CRP with all-cause mortality will provide additional evidence.

Randomized controlled trials are the best way for establishing causality clinically, however, they may be difficult to conduct. In addition, confounding and reverse causation are inherent risks with traditional observational studies. Two-sample mendelian randomization (TSMR) is a type of instrumental variable (IVs) analysis that uses genetic variants as IVs to quantify causality [11, 12]. Recently, TSMR method has gained popularity in clinical research as a result of its capacity to overcome the effects of potential confounding and reverse causality [13]. In the present study, we also utilized a TSMR approach to evaluate the relationship between CRP and telomere length (TL), which served as a surrogate indicator for mortality [14, 15].

Based on the China Health and Retirement Longitudinal Study (CHARLS), we investigated the longitudinal trends in hs-CRP including temporal trends and cumulative levels, with risk of all-cause mortality. To further our understanding, we also employed a TSMR method to investigate the causal link between CRP and mortality. These can provide certain evidence for the future prediction of death in the community using hs-CRP.

## Methods

### Nationwide prospective cohort study

The data used in this study is obtained from the CHARLS [16]. CHARLS is a prospective survey aiming at the Chinese population aged 45 and above, covering 28 provinces, including 150 regions and 450 villages/communities. CHARLS conducted final CAPI interviews with 10,257 families, achieving a response rate of 80.5%. The study employed a multi-stage cluster random sampling method, and covered a broad geographical area across most of China’s provinces, suggesting that the study population can largely represent the total population of China aged 45 and older [16]. This survey mainly employed standardized questionnaires to collect socio-demographic information, lifestyle, and health-related data. Baseline data was collected through computer-assisted face-to-face interviews in 2011 (wave 1), followed by subsequent surveys in 2013 (wave 2), 2015 (wave 3), 2018 (wave 4), and 2020 (wave 5). In the CHARLS, blood samples were drawn in the year 2011 and 2015.

**Fig. 1:**
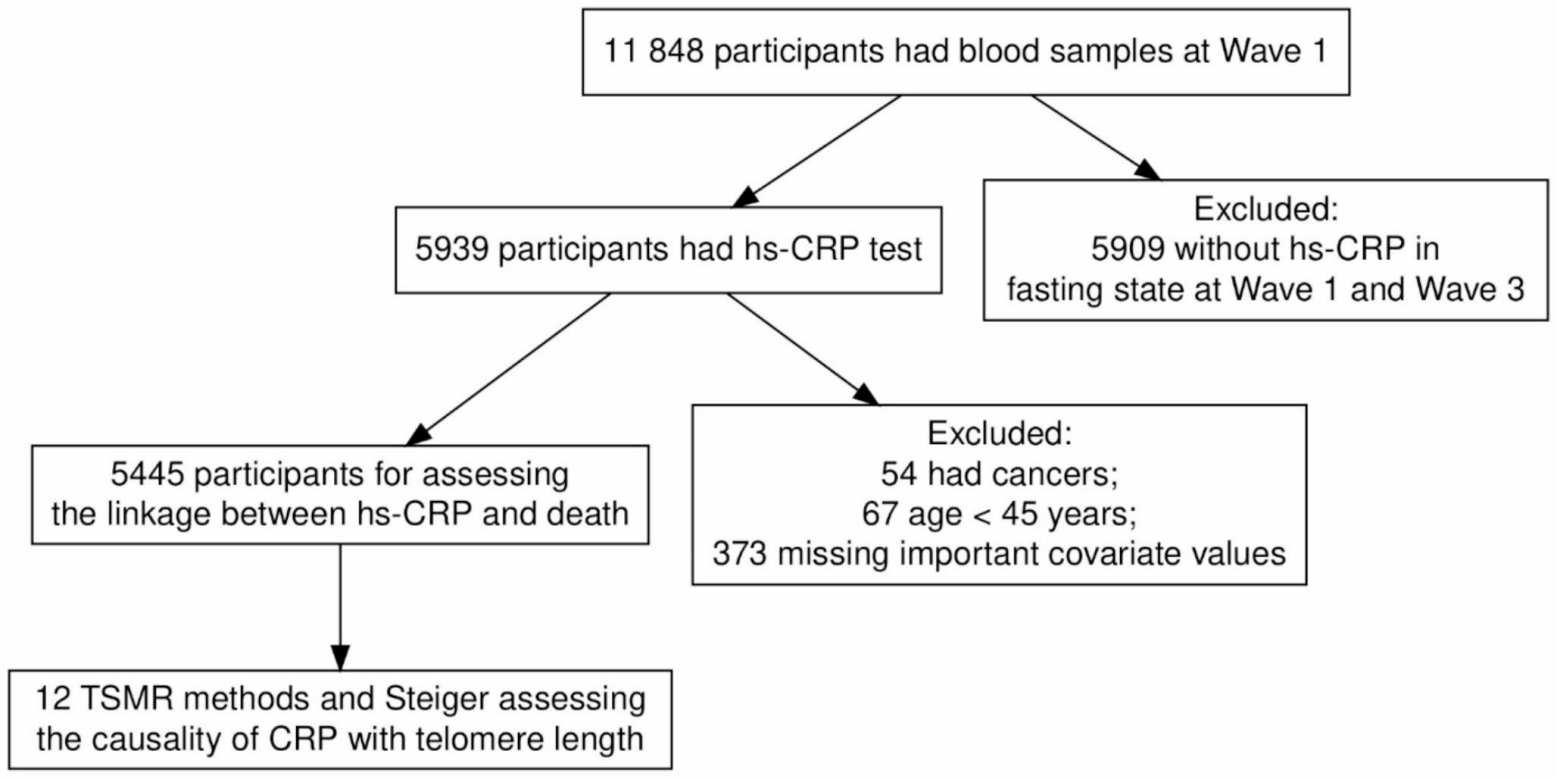
Flowchart of the study population. hs-CRP: high-sensitivity C-reactive protein; TSMR: two-sample mendelian randomization

Our study focused on the individuals over 45 years old in the CHARLS, covering the 2011–2018 period. Eligible participants were required to have documented hs-CRP levels at both wave 1 and wave 3, along with survival records from the year 2018. A total of 11,848 participants underwent baseline blood examinations in 2011. Below are the exclusion criteria: (1) age less than 45 years old; (2) missing important baseline covariates; (3) subjects with cancer. (Fig. 1)

CHARLS obtained approval from the Ethics Committee of Peking University, and informed consent forms were signed by all participants.

### Definition of hs-CRP

This investigation was centered on the dynamics of hs-CRP as the principal exposure. Our analysis considered both the longitudinal variations of hs-CRP over time and the cumulative hs-CRP (cumhs-CRP) levels, to provide a comprehensive view of its role in the population. Hs-CRP measurements were obtained using the immunoturbidimetric method and its concentration below 2 mg/L is generally indicative of low-grade systemic inflammation [17]. Following this criterion, we stratified this population from a longitudinal cohort (2011–2015) into four distinct trajectories [6]: Class 1: persistently low (low-low), Class 2: increasing from low to high (low-high), Class 3: decreasing from high to low (high-high), and Class 4: persistently high (high-high). For example, Class I represents those who had an hs-CRP measurement less than 2 mg/L at baseline and remained less than 2 mg/L at follow-up.

To assess cumulative inflammatory load, we employed a cumhs-CRP index using time-weighted averaging. The cumhs-CRP was calculated as follows: cumhs-CRP = [(hs-CRP2012 + hs-CRP2015) / 2] x (time2 - time1), where hs-CRP2012 is the hs-CRP level at the first measurement (wave 1), hs-CRP2015 is the level at a subsequent measurement (wave 3), and (time2 - time1) represents the duration between these two assessments.

### Definition of death

Death data were systematically collected for participants under observation from 2015 to 2018, with the occurrence of death being recorded as the outcome. To ascertain the vital status of participants over the study period, interviewers employed a computer-assisted personal interviewing system during onsite visitations. In the instances where a participant was deceased, the interviewers were tasked with obtaining the pertinent details by conducting interviews with the household members of the deceased.

### Determination of covariates

In this study, trained investigators conducted a thorough questionnaire survey with the objective of collecting detailed data on the demographic characteristics, lifestyle, and various health conditions of the participants. The survey encompassed a range of information, including demographic characteristics (age, gender, marital status, and education level, residence), lifestyle (smoking and drinking history), as well as measurements of blood pressure and body mass index (BMI, kg/m2). Additionally, various health conditions were assessed, and the study focused on self-reported, medical conditions diagnosed by physicians, including hypertension, dyslipidemia, diabetes, cardiovascular diseases, stroke, kidney disease, liver disease, and digestive diseases. These various health conditions’ definitions have been reported in prior studies [18]. Information on medication use for these conditions was also all collected and considered as important covariates. In the CHARLS, blood samples were gathered by trained Chinese Center for Disease Control (CCDC) staff according to standardized procedures, with participants required to fast prior to sampling. Subsequently, all samples were promptly frozen and transferred to the CCDC in two weeks, where detailed biochemical analysis was conducted at the Capital Medical University laboratories. The following indicators were considered as covariates: fasting glucose, glycated hemoglobin, low-density lipoprotein cholesterol (LDL-c), triglycerides, high-density lipoprotein cholesterol (HDL-c), total cholesterol, hemoglobin, and serum creatinine.

### Statistical analysis

The statistical analysis for this study was conducted using Stata 16.0 and R version 4.2. Continuous variables with a normal distribution were presented as the mean ± standard deviation, and group comparisons were conducted utilizing one-way analysis of variance (ANOVA). For continuous variables that did not display a normal distribution, we performed group comparisons using the Kruskal-Wallis H test. Categorical variables were expressed as counts and/or percentages, and differences between groups were assessed using the chi-square test.

We strive to ensure the completeness of the data we have screened. In fact, except for SBP (55 individuals, 1%), DBP (62 individuals, 1.1%), glycated hemoglobin (24 individuals, 0.4%), and hemoglobin (46 individuals, 0.8%) having missing values, there are no missing values for other variables. In the original analysis, we did not deal with the missing values, and we will conduct multiple imputation analysis later on.

Prior to conducting multivariate regression analysis, a thorough evaluation for multicollinearity was undertaken using the variance inflation factor (VIF). Then we employed stepwise regression analysis to select models with the lowest Akaike information criterion (AIC) to ensure the inclusion of the most appropriate variables.

This study employed binary logistic regression analysis with Class 1 as the reference to investigate the relationship between dynamic changes in hs-CRP levels and the mortality risk. Three models were developed in the primary analysis. After stepAIC selection, model 1 incorporated covariates including age, gender, education, smoking, and drinking, and model 2 extended the model 1 by incorporating diastolic blood pressure, HDL-c, and fasting glucose. Subsequent model 3 was adjusted for variables present in the model 2, with digestive disease being the additional factor considered. Additionally, we also conducted a comprehensive subgroup analysis. The following subgroups were considered: age (≥ 60 years or < 60 years), gender, marital status, education, residence, smoking, drinking, hypertension, diabetes, dyslipidemia, cardiovascular diseases, and stroke. In the subgroup analysis, the interactions between longitudinal changes in hs-CRP levels and the aforementioned factors were also assessed.

Additionally, cumhs-CRP levels were also categorized into quartiles, with Q1 as the reference, after stepAIC selection, a binary logistic regression analysis was employed to evaluate the relationship of cumhs-CRP quartiles with mortality, adjusting for age, education, gender, smoking, drinking, diastolic blood pressure, glucose, HDL-c, and digestive diseases. Moreover, we also examined potential non-linear associations between cumhs-CRP quartiles and mortality using restricted cubic splines (RCS). When interpreting the results of an RCS analysis, the 50th percentile value of the predictor variable was selected as the reference point. Cumhs-CRP levels were divided into two distinct segments based on this inflection point. This segmented logistic regression allowed for a more nuanced understanding of the relationship between these two variables in each segment.

We conducted three sensitivity analyses to validate our findings: (1) The primary analysis was replicated utilizing multiple imputation method; (2) The associations of baseline hs-CRP measured in the year 2012 and 2015, with the mortality risk were evaluated; (3) We also examined the relationship between the fluctuations in longitudinal hs-CRP levels and the mortality from the year 2020.

### Two-sample mendelian randomization

Based on a TSMR design, we analyzed the causal relationship of CRP with TL with inverse variance weighted (IVW) method. The summary data for CRP comprised both the discovery and validation set. The discovery set was obtained from the cohorts for HARGE Consortium, which is the largest genome-wide association study (GWAS) on CRP, including 575,531 subjects [19]. The validation set was derived from the pooled data from UKB bank, which included 389,057 subjects, with a total of 10,783,679 SNP loci [20]. The pooled data for TL were obtained from a large GWAS study involving 472,174 cases and 20,134,421 SNP loci of European ancestry (ieu-b-4879). First, candidate IVs with *P* < 5∗10 − 8 at genome-wide significance threshold were included. The criterion for linkage disequilibrium threshold was then set to r2 < 0.001 with a genetic distance of 10,000 kb after clumping with PLINK. Second, we explored if the aforementioned SNPs were linked to known confounders using the Catalog and PhenoScanner, if so, the SNP was excluded. Additionally, if F statistic > 10, weak IV is generally considered unbiased. We also used other approaches for further identifying these findings’ robustness. MR Egger intercept was employed to assess the pleiotropy, and the “leave-one-out” method and Cochran Q statistic was used to investigate potential heterogeneities.

### Feature interpretation and visualization of mortality

We also employed the Extreme Gradient Boosting (XGBoost) algorithm and SHapley Additive exPlanations (SHAP) for identifying determinants of mortality risk. A game-theoretic approach to machine learning, SHAP assigns an importance value to each feature that represents its contribution to the predictions [21]. And the SHAP important plot and heat force plot allowed us to visualize which variables are most important to all-cause mortality as well as how they influence it.

## Results

### Baseline characteristics of study participants

During the follow-up period from 2015 to 2018, there were 233 deaths among 5445 participants, resulting in a mortality proportion of 4.28%. Participants’ baseline characteristics are presented in Table 1. Overall, there were significant differences observed in hs-CRP in 2012, hs-CRP in 2015, and cumhs-CRP levels in Class 2, 3 and 4 in comparison to Class 1. And there are also significant differences in all-cause mortality among different classes. A similar trend was noted in age, gender, marital status, BMI, smoking, drinking, systolic and diastolic blood pressure, hemoglobin, glucose, triglycerides, LDL-c, HDL-c, and serum creatinine levels. Additionally, different classes also differed significantly in terms of the prevalence of certain comorbid conditions like hypertension, dyslipidemia, diabetes, cardiovascular diseases, lung and digestive diseases.

**Table 1:** Baseline characteristics of study population according to dynamitic changes in the hs-CRP

Characteristic	Dynamitic changes in the hs-CRP					*p*-value^b^
	Overall, (*N* = 5,445) ^a^	Class 1 (*N* = 2,835) ^a^	Class 3 (*N* = 566) ^a^	Class 2 (*N* = 1,196) ^a^	Class 4, (*N* = 848) ^a^	
**Age** (years)	59 ± 9	59 ± 9	60 ± 9	59 ± 9	61 ± 9	< 0.001
**Gender** (%)						< 0.001
Female	2,932 (54%)	1,542 (54%)	260 (46%)	661 (55%)	469 (55%)	
Male	2,513 (46%)	1,293 (46%)	306 (54%)	535 (45%)	379 (45%)	
**Residence** (%)						0.205
Rural	4,640 (85%)	2,426 (86%)	478 (84%)	1,031 (86%)	705 (83%)	
Urban	803 (15%)	408 (14%)	88 (16%)	164 (14%)	143 (17%)	
**Education** (%)						0.218
Illiterate	1,564 (29%)	803 (28%)	158 (28%)	336 (28%)	267 (31%)	
Middle school or below	3,391 (62%)	1,761 (62%)	351 (62%)	760 (64%)	519 (61%)	
Middle school higher	489 (9%)	271 (10%)	57 (10%)	99 (8%)	62 (7%)	
**Marital status** (%)						0.003
Married	4,653 (85%)	2,450 (86%)	470 (83%)	1,037 (87%)	696 (82%)	
Other	792 (15%)	385 (14%)	96 (17%)	159 (13%)	152 (18%)	
**Smoking** (%)						0.011
No Yes	3,335 (61%) 2,110 (39%)	1,759 (62%) 1,076 (38%)	313 (55%) 253 (45%)	753 (63%) 443 (37%)	510 (60%) 338 (40%)	
**Drinking** (%)						< 0.001
No	3,648 (67%)	1,870 (66%)	345 (61%)	819 (68%)	614 (72%)	
Yes	1,797 (33%)	965 (34%)	221 (39%)	377 (32%)	234 (28%)	
**Body Mass Index** (kg/m^2^)	23.7 ± 3.6	23.1 ± 3.3	23.5 ± 3.6	24.0 ± 3.4	25.1 ± 4.1	< 0.001
**Systolic blood pressure** (mmHg)	130 ± 21	128 ± 20	130 ± 21	131 ± 21	135 ± 23	< 0.001
**Diastolic blood pressure** (mmHg)	76 ± 12	74 ± 12	75 ± 12	77 ± 12	78 ± 12	< 0.001
**Hemoglobin** (g/L)	14.30 (13.10, 15.50)	14.20 (13.00, 15.40)	14.30 (13.20, 15.50)	14.40 (13.10, 15.80)	14.30 (13.20, 15.70)	0.007
**Fasting glucose** (mg/dL)	102 (95, 112)	101 (94, 110)	102 (95, 114)	103 (95, 114)	105 (97, 118)	< 0.001
**Serum creatinine** (mg/dL)	0.75 (0.64, 0.87)	0.73 (0.64, 0.86)	0.76 (0.66, 0.90)	0.76 (0.64, 0.87)	0.77 (0.67, 0.89)	< 0.001
**Total cholesterol** (mg/dL)	194 ± 38	193 ± 36	188 ± 39	197 ± 38	200 ± 41	< 0.001
**Triglycerides** (mg/dL)	105 (74, 152)	96 (71, 138)	94 (69, 136)	119 (81, 178)	126 (88, 186)	< 0.001
**HDL-c** (mg/dL) ^c^	51 ± 15	53 ± 15	51 ± 15	49 ± 15	46 ± 14	< 0.001
**LDL-c** (mg/dL) ^c^	118 ± 34	118 ± 33	115 ± 34	116 ± 35	121 ± 39	0.002
**Hba1c** (%)	5.20 (4.90, 5.40)	5.10 (4.90, 5.40)	5.20 (4.90, 5.50)	5.20 (4.90, 5.40)	5.20 (4.90, 5.60)	< 0.001
**CRP2012** (mg/L)	1.01 (0.55, 2.09)	0.65 (0.42, 1.03)	3.43 (2.48, 5.79)	0.96 (0.61, 1.38)	3.74 (2.69, 6.35)	< 0.001
**CRP2015** (mg/L)	1.50 (0.80, 2.70)	0.90 (0.60, 1.30)	1.10 (0.70, 1.50)	3.00 (2.40, 5.00)	3.80 (2.70, 6.70)	< 0.001
**CumCRP** (mg/L)	4 (2, 8)	2 (2, 3)	7 (5, 11)	6 (5, 9)	13 (9, 21)	< 0.001
**Hypertension** (%)						< 0.001
No	4,026 (74%)	2,224 (79%)	414 (73%)	862 (72%)	526 (62%)	
Yes	1,397 (26%)	599 (21%)	150 (27%)	331 (28%)	317 (38%)	
**Dyslipidemia** (%)						< 0.001
No	4,785 (90%)	2,554 (92%)	503 (90%)	1,025 (88%)	703 (85%)	
Yes	553 (10%)	229 (8%)	53 (10%)	143 (12%)	128 (15%)	
**Diabetes** (%)						< 0.001
No	5,076 (94%)	2,670 (95%)	528 (94%)	1,115 (94%)	763 (91%)	
Yes	326 (6%)	140 (5%)	36 (6%)	72 (6%)	78 (9%)	
**Heart problem** (%)						0.004
No	4,791 (88%)	2,531 (89%)	497 (88%)	1,044 (87%)	719 (85%)	
Yes	654 (12%)	304 (11%)	69 (12%)	152 (13%)	129 (15%)	
**Stroke** (%)						0.450
No	5,340 (98%)	2,786 (98%)	551 (97%)	1,174 (98%)	829 (98%)	
Yes	105 (2%)	49 (2%)	15 (3%)	22 (2%)	19 (2%)	
**Kidney diseases** (%)						0.433
No	5,099 (94%)	2,660 (94%)	533 (94%)	1,108 (93%)	798 (94%)	
Yes	346 (6%)	175 (6%)	33 (6%)	88 (7%)	50 (6%)	
**Lung diseases** (%)						< 0.001
No	4,880 (90%)	2,602 (92%)	495 (87%)	1,048 (88%)	735 (87%)	
Yes	565 (10%)	233 (8%)	71 (13%)	148 (12%)	113 (13%)	
**Liver diseases** (%)						0.623
No	5,228 (96%)	2,722 (96%)	538 (95%)	1,151 (96%)	817 (96%)	
Yes	217 (4%)	113 (4%)	28 (5%)	45 (4%)	31 (4%)	
**Digestive diseases** (%)						0.028
No	4,170 (77%)	2,138 (75%)	449 (79%)	908 (76%)	675 (80%)	
Yes	1,275 (23%)	697 (25%)	117 (21%)	288 (24%)	173 (20%)	
**Hypertension diseases medications** (%)						< 0.001
No	4,406 (81%)	2,409 (85%)	453 (80%)	949 (79%)	595 (70%)	
Yes	1,039 (19%)	426 (15%)	113 (20%)	247 (21%)	253 (30%)	
**Dyslipidemia medications** (%)						< 0.001
No	5,158 (95%)	2,730 (96%)	539 (95%)	1,116 (93%)	773 (91%)	
Yes	287 (5%)	105 (4%)	27 (5%)	80 (7%)	75 (9%)	
**Diabetes medications** (%)						0.002
No	5,266 (97%)	2,755 (97%)	550 (97%)	1,159 (97%)	802 (95%)	
Yes	179 (3%)	80 (3%)	16 (3%)	37 (3%)	46 (5%)	
**Heart diseases medications** (%)						0.027
No	5,071 (93%)	2,657 (94%)	533 (94%)	1,110 (93%)	771 (91%)	
Yes	374 (7%)	178 (6%)	33 (6%)	86 (7%)	77 (9%)	
**Stroke medications** (%)						0.114
No	5,374 (99%)	2,802 (99%)	553 (98%)	1,184 (99%)	835 (98%)	
Yes	71 (1%)	33 (1%)	13 (2%)	12 (1%)	13 (2%)	
**Kidney diseases medications** (%)						0.655
No	5,320 (98%)	2,773 (98%)	555 (98%)	1,163 (97%)	829 (98%)	
Yes	125 (2%)	62 (2%)	11 (2%)	33 (3%)	19 (2%)	
**Lung diseases medications** (%)						0.999
No	5,444 (100%)	2,834 (100%)	566 (100%)	1,196 (100%)	848 (100%)	
Yes	1 (0%)	1 (0%)	0 (0%)	0 (0%)	0 (0%)	
**Liver diseases medications** (%)						0.842
No	5,371 (99%)	2,797 (99%)	556 (98%)	1,181 (99%)	837 (99%)	
Yes	74 (1%)	38 (1%)	10 (2%)	15 (1%)	11 (1%)	
**Digestive diseases medications** (%)						0.035
No	4,725 (87%)	2,431 (86%)	502 (89%)	1,036 (87%)	756 (89%)	
Yes	720 (13%)	404 (14%)	64 (11%)	160 (13%)	92 (11%)	
**Death** (%)						< 0.001
Alive	5,212 (96%)	2,756 (97%)	535 (95%)	1,138 (95%)	783 (92%)	
Died	233 (4%)	79 (3%)	31 (5%)	58 (5%)	65 (8%)	

^a^Mean ± SD; n (%); Median (IQR)

^b^One-way ANOVA; Pearson’s Chi-squared test; Kruskal-Wallis rank sum test; Fisher’s exact test

^c^ HDL-c, high density lipoprotein cholesterol; LDL-C, low density lipoprotein cholesterol

### Association between changes of hs-CRP and all-cause mortality

The association between the two is detailed in Table 2. We identified relevant variables through multicollinearity analysis and stepAIC selection. In Model 3, after accounting for potential factors, compared to Class 1, the ORs (95% CIs) were 1.79 (1.24 to 2.56) in Class 2, 1.78 (1.13 to 2.75) in Class 3, and 2.34 (1.62 to 3.36) in Class 3, respectively, which is essentially consistent with the findings from Model 1 and Model 2.

**Table 2:** Association between dynamic changes of hs-CRP levels and all-cause mortality

Characteristic	Model 1			Model 2			Model 3		
	OR	95% CI	*p*-value	OR^1^	95% CI	*p*-value	OR	95% CI	*p*-value
Changes of hs-CRP									
Class 1 **(52.1%)**	—	—		—	—		—	—	
Class 2 **(10.4%)**	1.82	1.27, 2.61	0.001	1.79	1.24, 2.57	0.002	1.79	1.24, 2.58	0.002
Class 3 **(22.0%)**	1.79	1.14, 2.76	0.009	1.79	1.14, 2.76	0.010	1.78	1.13, 2.75	0.011
Class 4 **(15.5%)**	2.47	1.73, 3.51	< 0.001	2.36	1.64, 3.38	< 0.001	2.34	1.62, 3.36	< 0.001

Model 1: adjusted for age, gender, education, smoking, and drinking

Model 2: adjusted for age, gender, education, smoking, drinking, diastolic blood pressure, high density lipoprotein-cholesterol, and fasting glucose.

Model 3: adjusted for age, gender, education, smoking, drinking, diastolic blood pressure, high density lipoprotein-cholesterol, fasting glucose, and digestive diseases.

### L-shaped relationship between cumhs-CRP and all-cause mortality

**Fig. 2:**
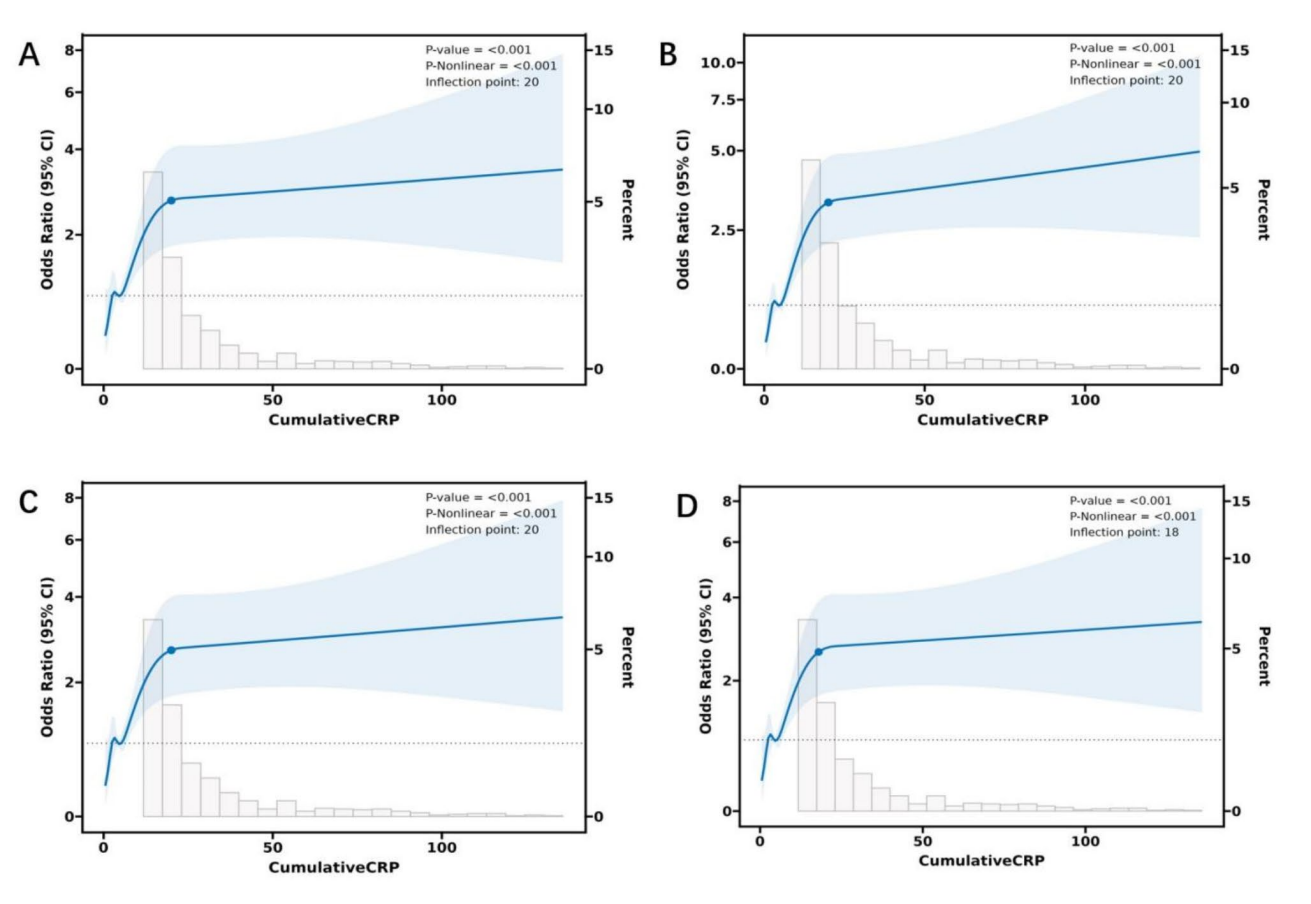
Association between CumulativeCRP and all-cause mortality with the RCS function. Model with 4 knots located at 5th, 35th, 65th and 95th percentiles. Y-axis represents the OR to present all-cause mortality for any value of cumulativeCRP compared to individuals with reference value (50th percentile) of cumulativeCRP. The logistic regression was adjusted for age and gender. **A** displayed the model of uncorrected latent variables. The logistic regression of model 1 was adjusted for age, gender, education, smoking and drinking (**B**). The logistic regression of model 2 was adjusted for age, gender, education, smoking, drinking, diastolic blood pressure, fasting glucose, and HDL- cholesterol (**C**). The logistic regression of model 3 was adjusted for age, gender, education, smoking, drinking, diastolic blood pressure, fasting glucose, HDL- cholesterol and digestive diseases (**D**)

Before undertaking this analysis, we also selected relevant variables using multicollinearity analysis and stepwise AIC. After adjusting for potential factors, the results indicated a significant link between cumhs-CRP levels and the risk of all-cause mortality (OR 1.15, 95% CI 1.09–1.21, *p* < 0.001) (Table 3). In order to delve into their dose-response relationship, we stratified cumhs-CRP levels into four quartiles and analyzed the death risk across these quartiles. The findings indicated that, in contrast to the Q1, the ORs for Q3 and Q4, along with their corresponding 95% CIs, were 1.66 (1.05 to 2.69) and 3.03 (1.98 to 4.75), respectively (Table 3). Furthermore, we identified an “L-shaped” relationship between the two, with the inflection point at a hs-CRP value of 20 mg/L. This finding led us to divide the study population into two cohorts: one with hs-CRP levels below 20 mg/L and another with levels at or above this value. Subsequent segmented regression analyses were applied to each cohort, with the outcomes delineated in Fig. 2 and Supplementary Table S1.

**Table 3:** Association between cumhs-CRP according to quartiles and all-cause mortality

Characteristic	*N*	Event *N*	OR^a^	95% CI^a^	*p*-value
**Cumhs-CRP** Cumhs-CRP (adjusted)*	5445	228	1.15	1.09, 1.21	< 0.001
Q1 [0.33 mg/L, 2.38 mg/L]	1,339	30	—	—	
Q2 [2.38 mg/L, 4.28 mg/L]	1,358	41	1.29	0.79, 2.13	0.305
Q3 [4.28 mg/L, 7.85 mg/L]	1,345	53	1.66	1.05, 2.69	0.034
Q4 [7.85 mg/L, 72 mg/L]	1,342	104	3.03	1.98, 4.75	< 0.001

^a^OR = Odds Ratio, CI = Confidence Interval

* adjusted for age, education, gender, smoking, drinking, fasting glucose, diastolic blood pressure, high density lipoprotein-cholesterol, and digestive diseases medications

### Subgroup analysis

We conducted detailed subgroup analyses by categorizing participants based on various factors including age, gender, marital status, residence, education, smoking and drinking, as well as the presence of hypertension, diabetes, dyslipidemia, stroke, and cardiovascular diseases. The comprehensive results are presented in Table 4. In most subgroups, such as the elderly, males, married individuals, rural residents, smokers, and drinkers, participants in other classes had a higher risk of all-cause mortality than those in Class 1, as outlined in Table 4. Additionally, our analysis revealed no significant interactions between these above factors and the changes of hs-CRP levels (p-values for interaction > 0.05).

**Table 4:** Associations of different classes of change in hs-CRP with mortality stratified according to different factors

	Dynamic changes in CRP levels, OR (95%CI)				
Subgroup	Class 1	Class 2	Class 3	Class 4	*P* for interaction
**Age (years)**					0.653
<60 ≥60	Reference Reference	1.41 (0.79, 2.48) 2.15 (1.33, 3.46)	1.84 (0.93, 3.47) 1.66 (0.89, 2.96)	2.04 (1.12, 3.64) 2.51 (1.57, 3.99)	
**Gender**					0.258
Male Female	Reference Reference	2.17 (1.40, 3.34) 1.19 (0.58, 2.32)	1.84 (1.07, 3.10) 1.63 (0.70, 3.51)	2.86 (1.84, 4.41) 1.53 (0.77, 2.96)	
**Marital status**					0.204
Married Other	Reference Reference	1.79 (1.17, 2.70) 1.86 (0.89, 3.79)	2.23 (1.35, 3.59) 0.84 (0.24, 2.29)	2.49 (1.63, 3.77) 2.43 (1.21, 4.81)	
**Residence**					0.305
Urban Rural	Reference Reference	0.65 (0.18, 1.83) 2.04 (1.41, 2.95)	1.25 (0.35, 3.55) 2.21 (1.37, 3.47)	1.15 (0.40, 2.89) 3.37 (2.40, 4.86)	
**Education level**					0.347
Illiterate Middle school or below Middle school higher	Reference Reference Reference	1.86 (1.00, 3.41) 1.95 (1.23, 3.08) NA	1.39 (0.59, 3.02) 2.14 (1.23, 3.62) NA	1.55 (0.80, 2.93) 3.06 (1.95, 4.79) NA	
**Smoking**					0.103
No Yes	Reference Reference	1.34 (0.75, 2.35) 2.21 (1.37, 3.56)	1.72 (0.83, 3.35) 1.86 (1.03, 3.27)	1.37 (0.73, 2.53) 3.21 (2.03, 5.08)	
**Drinking**					0.054
No Yes	Reference Reference	1.27 (0.78, 2.04) 3.28 (1.81, 5.99)	1.97 (1.12, 3.36) 1.65 (0.73, 3.51)	1.90 (1.20, 2.98) 3.49 (1.85, 6.56)	
**Hypertension**					0.052
No Yes	Reference Reference	2.46 (1.57, 3.82) 0.89 (0.44, 1.69)	2.04 (1.15, 3.50) 1.27 (0.58, 2.62)	2.98 (1.88, 4.71) 1.40 (0.76, 2.53)	
**Dyslipidemia**					0.321
No Yes	Reference Reference	1.96 (1.33, 2.87) 0.69 (0.20, 2.02)	1.78 (1.09, 2.83) 1.64 (0.47, 5.03)	2.59 (1.76, 3.79) 0.66 (0.17, 2.09)	
**Diabetes**					0.631
No Yes	Reference Reference	1.87 (1.31, 2.68) 0.76 (0.16, 2.69)	2.03 (1.28, 3.14) 1.68 (0.36, 6.15)	3.13 (2.20, 4.45) 0.97 (0.25, 3.17)	
**Heart diseases**					0.393
No Yes	Reference Reference	2.02 (1.37, 2.99) 0.81 (0.25, 2.30)	1.89 (1.14, 3.03) 1.31 (0.38, 3.92)	2.52 (1.69, 3.75) 1.64 (0.63, 4.16)	
**Stroke**					0.061
No Yes	Reference Reference	1.77 (1.25, 2.51) 2.29 (0.09, 59.66)	1.79 (1.13, 2.76) NA	2.81 (1.99, 3.95) NA	

### Sensitivity analyses

To substantiate the robustness of our principal analysis, we re-evaluated these relationships, employing diverse exposure, outcome, and statistical approaches. In alignment with the primary analysis, we also conducted multicollinearity assessments and employed stepAIC to filter pertinent variables prior to multivariable analysis. In the first sensitivity analysis, using the natural logarithm-transformed hs-CRP from 2012 as the exposure variable, we found a significant association with all-cause mortality in the cohort (OR = 1.21, 95% Cl 1.07 to 1.36, *p* = 0.002) (Supplementary Table S2). Additionally, with lnhs-CRP from 2015 as the exposure variable, similar results were observed (OR 1.56, 95% CI 1.38 to 1.75, *p* < 0.001) (Supplementary Table S3), which was similar to the results of another sensitivity analysis (Supplementary Table S4). In the second sensitivity analysis, mortality from 2020 served as the outcome. The findings suggested that in comparison to Class 1, the adjusted ORs (95% CIs) for mortality risk were 1.41 (1.05 to 1.87) for Class 2, 1.65 (1.16 to 2.30) for Class 3, and 1.87 (1.40 to 2.50) for Class 4 (Supplementary Table S5). Finally, multiple imputation of the original data also confirmed that the different classes of hs-CRP changes remained significantly associated with a heightened risk of mortality (OR = 1.85 for Class 2, OR = 1.76 for Class 3, and OR = 2.41 for Class 4). (Supplementary Table S6)

### Two-sample mendelian randomization

In the discovery set, results from IVW approach showed that genetically predicted CRP was positively associated with TL (**β**= -0.043, 95% CI -0.058 to -0.027, *P* = 1.580-7), which was also validated by other MR analyses. Similarly, in the validation set, the results from the random effects IVW method demonstrated a negative association between CRP and TL (**β**= -0.038, 95% CI -0.062 to -0.014, *P* = 0.001), which was also supported by other sensitive analyses. The “leave-one-out” analysis revealed that the IVs used in this study had no significant effect on the results, indicating significant confidence in the findings. SNPs were found to be symmetrically distributed in funnel plots, suggesting that potential bias is unlikely to affect causality. (Table 5)

**Table 5:** Causality of C-reactive protein with telomere length

Exposure	Outcome	nSNP	Beta	SE	*P* value	MR Steiger	Pleiotropy	Heterogeneity
ebi-a-GCST 90014002^a^	ieu-b-4879	211	-0.038	0.0121	0.0017	True	0.083	<0.001
ebi-a-GCST 90029070^b^	ieu-b-4879	218	-0.0425	0.0081	1.580E-07	True	0.068	<0.001

^a^ Pubmed ID:35459240; ^b^ Pubmed ID: 34017140

### Feature interpretation and visualization of mortality

**Fig. 3:**
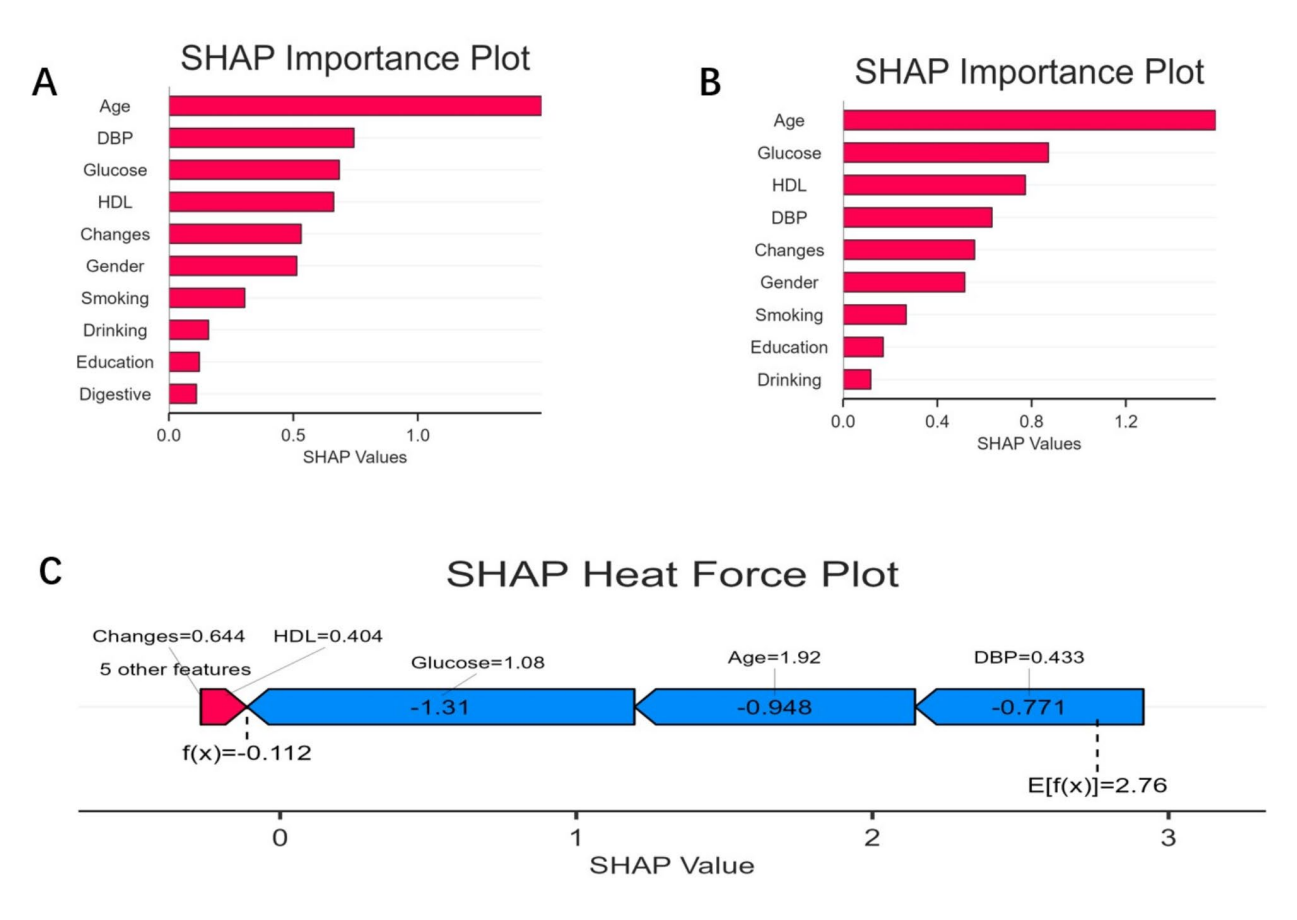
**A** and **B** are both SHAP importance plots, showing the most crucial features for death prediction respectively. **C** is a SHAP heat force plot, displaying changes in hs-CRP as a critically important predictor for all-cause mortality

SHAP values revealed each feature’s contribution to the prediction of the three models, as shown in Fig. 3. The longest bars belonged to features such as age, dbp, fasting glucose, HDL-c and changes of hs-CRP, which were more crucial to the prediction of the models.

## Discussion

Utilizing the CHARLS dataset, we conducted a prospective cohort of participants over an extended period and discerned a marked link between dynamic changes in hs-CRP levels and the risk of all-cause mortality. An additional analysis revealed that the cumhs-CRP quartiles are tied to the mortality risk in a non-linear manner. The validity of these associations was also further confirmed through sensitivity analyses and TSMR.

Epidemiological evidence has consistently demonstrated a significant link between baseline CRP levels, especially those in the upper quartiles, and the likelihood of all-cause mortality [6–9], which are consistent with our findings. However, there is limited research on the longitudinal association of CRP in predicting mortality risk in low- and middle-income countries. A total of 9253 participants from the PREVEND and the Framingham Heart Study, all of whom had CRP measurements at two different time points, were included. The findings suggested that an increase in CRP over time can predict overall mortality in the general population (HR 1.10; 95% CI 1.05 to 1.16) [6]. Analogous findings have also been described in CRONICAS study, and the authors observed a robust linkage between cumhs-CRP and overall mortality, with cumhs-CRP measurements proving more effective for predicting mortality risk compared to than single baseline measurements [7]. Additionally, a study focusing on the Korean population, a high-income demographic in East Asia, reported significantly lower CRP levels compared to European populations. Although baseline CRP levels showed some correlation with all-cause mortality among Korean males, cumCRP did not exhibit such a relationship [22]. These divergent results may be attributed to differences in study design, sample selection, measurement techniques, and the biological variability of inflammatory markers. Overall, even a slight elevation in hs-CRP may exert lasting effects on one’s health. Therefore, the routine monitoring of hs-CRP levels is crucial for effectively preventing and controlling chronic conditions.

In our investigation, we employed the TSMR method to delve into the causality of between CRP with mortality risk. Considering the principle of “genetic-environmental equivalence”, we selected telomere length, a biomarker intricately linked to all-cause mortality, as the outcome variable in the MR analysis, rather than mortality itself [23]. The findings suggested a negative causality between CRP levels and TL, thereby indicating that higher concentrations of CRP are associated with reduced TL. A substantial body of research has demonstrated a closely link between reduced TL and heightened risks of all-cause mortality, particularly cardiac death [14, 15]. Given the limited number of cancer cases, we excluded patients with cancer. As a result, the majority of the all-cause mortality observed in our study may be likely attributable to cardiac deaths. Therefore, our findings further substantiated the causality of CRP with deaths other than cancer.

The biological mechanisms underlying chronic inflammation are highly complex, involving a multitude of cell types, signaling pathways, and molecular processes [24]. Persistent inflammatory responses can lead to tissue and organ damage, resulting in functional impairments and incremental mortality risk [25]. Additionally, numbers studies have elaborated chronic inflammation is associated with metabolic disorders and an elevated risk of cardiovascular diseases [26, 27]. Moreover, impairment of immune defenses due to chronic inflammation can diminish the body’s resistance to infections and neoplasms [28], thereby adversely affecting one’s health and lifespan. Emerging research further implicates chronic inflammation in the aging process, with potential ramifications for an individual’s healthspan and overall longevity [23, 29]. For instance, senescent cells, in particular, secrete pro-inflammatory mediators that can accelerate the progression of a variety of chronic conditions [30].

Our study boasted several strengths. Firstly, diverging from previous research that predominantly focusing on populations of high-income countries, our investigation extended its applicability to low- and middle-income individuals and regions. Additionally, our study sample included healthy individuals from diverse regions across China, demonstrating broad representativeness. Moreover, our findings supported the dynamic changes in hs-CRP as a valuable clinical biomarker for identifying high-risk mortality among the general population. Finally, we employed a TSMR approach to investigate the causal relationship between CRP and TL, a surrogate marker for mortality, further strengthening the credibility of our observational results.

However, this study had certain shortcomings. Firstly, we were unable to fully control for potential confounding factors including diet and lifestyle habits. In an effort to address the limitation, we specifically assessed the multicollinearity of variables and subsequently employed stepAIC selection. Additionally, we conducted three sensitivity analyses, all of which consistently reinforced our main findings. Secondly, we could not establish a causal relationship between them, but we further utilized TSMR to examinate the causality of CRP with TL. TL cannot fully represent all-cause mortality, but we have taken into account that interaction between genes and the environment in the event of death may be more complex, and TL may serve as a very objective biomarker reflecting health status and life expectancy. Furthermore, the small sample size of deaths in certain subgroups may have potentially impacted the robustness of findings within those subgroups.

In summary, we identified a significant relationship of the dynamic changes of hs-CRP levels with all-cause mortality risk in China, suggesting that regular monitoring of hs-CRP levels could potentially aid in the identification of individuals who are at a higher risk of mortality. Subsequent studies should further focus on the effective interventions to address chronic inflammation.

## Electronic supplementary material

Below is the link to the electronic supplementary material.

Supplementary Material 1

## Data Availability

A list of the online repositories with accession numbers can be found at: http://charls.pku.edu.cn/en.
