# Supplementary material for: Dynamic changes in hs-CRP and risk of all-cause mortality among middle-aged and elderly adults: findings from a nationwide prospective cohort and mendelian randomization
Source: Aging Clin Exp Res. 2024 Oct 26;36(1):210. doi: 10.1007/s40520-024-02865-w (PMC11512892; doi:10.1007/s40520-024-02865-w)
Supplement: Supplementary file 1 — Supplementary Material 1 [file 40520_2024_2865_MOESM1_ESM.docx]

Supplementary Table 1: Effect of standardized cumhs-CRP level on all-cause mortality: adjusted odds ratios from segmented logistic regression analysis

| **Characteristic** | **OR per SD** | **95% CI** | **p-value** |
| --- | --- | --- | --- |
| Cumhs-CRP (< 16) | 1.33 | 1.02, 1.76 | 0.041 |
| Cumhs-CRP (≥ 16) | 1.24 | 1.13, 1.36 | <0.001 |
| ORs were adjusted for age and gender | | | |
|  | | | |

Supplementary Table 2: Association between lnhs-CRP in 2012 and all-cause mortality

| **Characteristic** | **N** | **Event N** | **OR**^1^ | **95% CI**^1^ | **p-value** |
| --- | --- | --- | --- | --- | --- |
| LnCRP (adjusted)* | 5,384 | 228 | 1.210 | 1.070, 1.363 | 0.002 |
| ^1^OR = Odds Ratio, CI = Confidence Interval | | | | | |
| * adjusted for age, gender, education, smoking, drinking, diastolic blood pressure, high density lipoprotein-cholesterol, fasting glucose, and digestive diseases medications | | | | | |

Supplementary Table 3: Association between lnhs-CRP in 2015 and all-cause mortality

| **Characteristic** | **N** | **Event N** | **OR**^1^ | **95% CI**^1^ | **p-value** |
| --- | --- | --- | --- | --- | --- |
| LnCRP2015 (adjusted)* | 5,384 | 228 | 1.453 | 1.282, 1.647 | <0.001 |
| ^1^OR = Odds Ratio, CI = Confidence Interval | | | | | |
| * adjusted for age, gender, education, smoking, drinking, diastolic blood pressure, high density lipoprotein-cholesterol, fasting glucose, serum creatinine, and digestive diseases medications. | | | | | |

Supplementary Table 4: Association between lncumhs-CRP and all-cause mortality

| **Characteristic** | **N** | **Event N** | **OR**^1^ | **95% CI**^1^ | **p-value** |
| --- | --- | --- | --- | --- | --- |
| Lncumhs-CRP (adjusted)* | 5,384 | 228 | 1.151 | 1.094, 1.211 | <0.001 |
| ^1^OR = Odds Ratio, CI = Confidence Interval | | | | | |
| * adjusted for age, gender, education, smoking, drinking, diastolic blood pressure, high density lipoprotein-cholesterol, fasting glucose, serum creatinine, and digestive diseases medications | | | | | |

**Supplementary Table** 5: Association between changes of hs-CRP and all-cause mortality in 2020

| **Characteristic** | **N** | **Event N** | **OR**^1^ | **95% CI**^1^ | **p-value** |
| --- | --- | --- | --- | --- | --- |
| Changes of hs-CRP (adjusted)* |  |  |  |  |  |
| class 1 | 2,721 | 156 | — | — |  |
| class 2 | 1,145 | 89 | 1.410 | 1.054, 1.878 | 0.020 |
| class 3 | 546 | 58 | 1.645 | 1.163, 2.300 | 0.004 |
| class 4 | 802 | 100 | 1.872 | 1.397, 2.499 | <0.001 |
| ^1^OR = Odds Ratio, CI = Confidence Interval | | | | | |
| * adjusted for age, education, gender, smoking, drinking, fasting glucose, diastolic blood pressure, high density lipoprotein-cholesterol, and digestive diseases medications | | | | | |

**Supplementary Table 6:** Univariate and multivariate analysis between changes of hs-CRP and all-cause mortality

| Dependent: Death |  | Non-Event | Event | OR (univariable) | OR (multivariable) | OR (multiple imputation) |
| --- | --- | --- | --- | --- | --- | --- |
| Changes of hs-CRP | Class 1 | 2756 (97.2) | 79 (2.8) | - | - | - |
|  | Class 2 | 1138 (95.2) | 58 (4.8) | 1.778 (1.254-2.508) | 1.794 (1.242-2.578) | 1.85 (1.29-2.65) |
|  | Class 3 | 535 (94.5) | 31 (5.5) | 2.021 (1.303-3.062) | 1.780 (1.128-2.747) | 1.76 (1.13-2.74) |
|  | Class 4 | 783 (92.3) | 65 (7.7) | 2.896 (2.061-4.056) | 2.340 (1.623-3.361) | 2.41 (1.68-3.45) |

* adjusted for age, education, gender, smoking, drinking, fasting glucose, diastolic blood pressure, high density lipoprotein-cholesterol, and digestive diseases medications
